# Comparative Studies of the Venom of a New Taipan Species, *Oxyuranus temporalis*, with Other Members of Its Genus

**DOI:** 10.3390/toxins6071979

**Published:** 2014-07-02

**Authors:** Carmel M. Barber, Frank Madaras, Richard K. Turnbull, Terry Morley, Nathan Dunstan, Luke Allen, Tim Kuchel, Peter Mirtschin, Wayne C. Hodgson

**Affiliations:** 1 Monash Venom Group, Department of Pharmacology, Faculty of Medicine, Nursing and Health Sciences, Monash University, Clayton, Victoria 3168, Australia; E-Mail: carmel.barber@monash.edu; 2 Venom Science Pty Ltd, Tanunda, South Australia 5352, Australia; E-Mails: frankmadaras@esc.net.au (F.M.); peter@venomsupplies.com (P.M.); 3 SA Pathology, IMVS Veterinary Services, Gilles Plains, South Australia 5086, Australia; E-Mails: randcturnbull@bigpond.com (R.K.T.); tim.kuchel@sahmri.com (T.K.); 4 Adelaide Zoo, Adelaide, South Australia 5000, Australia; E-Mail: tmorley@zoossa.com.au; 5 Venom Supplies, Tanunda, South Australia, South Australia 5352, Australia; E-Mails: nathan@venomsupplies.com (N.D.); luke@venomsupplies.com (L.A.)

**Keywords:** *Oxyuranus temporalis*, taipan, antivenom, neurotoxicity, snake, venom

## Abstract

Taipans are highly venomous Australo-Papuan elapids. A new species of taipan, the Western Desert Taipan (*Oxyuranus temporalis*), has been discovered with two specimens housed in captivity at the Adelaide Zoo. This study is the first investigation of *O. temporalis* venom and seeks to characterise and compare the neurotoxicity, lethality and biochemical properties of *O. temporalis* venom with other taipan venoms. Analysis of *O. temporalis* venom using size-exclusion and reverse-phase HPLC indicated a markedly simplified “profile” compared to other taipan venoms. SDS-PAGE and agarose gel electrophoresis analysis also indicated a relatively simple composition. Murine LD_50_ studies showed that *O. temporalis* venom is less lethal than *O. microlepidotus* venom. Venoms were tested *in vitro*, using the chick biventer cervicis nerve-muscle preparation. Based on *t*_90_ values, *O. temporalis* venom is highly neurotoxic abolishing indirect twitches far more rapidly than other taipan venoms. *O. temporalis* venom also abolished responses to exogenous acetylcholine and carbachol, indicating the presence of postsynaptic neurotoxins. Prior administration of CSL Taipan antivenom (CSL Limited) neutralised the inhibitory effects of all taipan venoms. The results of this study suggest that the venom of the *O. temporalis* is highly neurotoxic *in vitro* and may contain procoagulant toxins, making this snake potentially dangerous to humans.

## 1. Introduction

The taipans (*Oxyuranus* genus) are highly venomous Australo-Papuan elapids consisting of the Inland Taipan (*O. microlepidotus*), Coastal Taipan (*O. scutellatus*) and Papuan Taipan (previously *O. s. canni*; now *O. scutellatus*). However, phylogenetic studies have shown that even though the Papuan Taipan has been considered a distinct subspecies to the Coastal Taipan, there are no significant differences between the populations [1,2]. More recently, a third distinct species of taipan (*O. temporalis*, Western Desert Taipan) has been discovered [3]. Due to the apparent remote geographical distribution of this species only a handful of specimens have been collected and studied. Therefore, limited data exists about the distribution, appearance, diet and genetic variation of this species (see [2,3,4] for further details). Currently there are two wild-caught specimens housed at the Adelaide Zoo in South Australia. The venom of *O. temporalis* has not been studied.

Taipan venoms contain a variety of components including pre- and post-synaptic neurotoxins. Paradoxin (*O. microlepidotus*, [5]), taipoxin (*O. scutellatus*, [6]) and cannitoxin (previously *O. s. canni*, [7]) are presynaptic neurotoxins isolated from taipan venoms consisting of three subunits (α, β and γ) and molecular masses of approximately 45–47 kDa [5,6,7,8]. Postsynaptic neurotoxins including oxylepitoxin-1 (*O. microlepidotus*, [9]), α-scutoxin 1 (*O. scutellatus*, [10]), α-oxytoxin 1 (previously *O. s. canni*, [10]), taipan toxin 1 (*O. scutellatus*, [11]) and taipan toxin 2 (*O. scutellatus*, [11]) have also been isolated. These short-chain postsynaptic neurotoxins consist of a single subunit, with molecular masses ranging from 6726 Da to 6789 Da, are similar to those isolated from other elapid venoms [12]. Taipan venoms also contain other components including natriuretic-like peptides [13,14], prothrombin activators [14,15,16,17], toxins that reversibly block calcium channels (*i.e*., taicatoxin, [14,18]), Kunitz-type plasma kallikrein inhibitors [14,19] and cysteine-rich secretory proteins (CRISP) [14].

Studies using the chick biventer cervicis nerve-muscle preparation (*i.e*., a skeletal muscle preparation) have shown that all taipan venoms have *in vitro* neurotoxic activity [7,8,20,21]. CSL taipan antivenom has also been shown to either delay or prevent the neurotoxicity of these venoms *in vitro* [20].

Taipan envenoming in humans results in a range of common clinical effects including neurotoxicity, venom-induced consumption coagulopathy and mild rhabdomyolysis [22,23]. Other effects, though rarely reported, include renal failure, thrombotic microangiopathy and haemolytic anaemia [22]. The clinical symptoms of *O. temporalis* envenoming remain unknown as there have been no documented bites.

This study, being the first investigation of *O. temporalis* venom, examined the neurotoxic effects, lethality, and biochemical properties of the venom in comparison to the more well studied taipan venoms. This study provides valuable insight into the venom components and the likely effects of human envenoming.

## 2. Results

### 2.1. Size-Exclusion Chromatography

**Figure 1:**
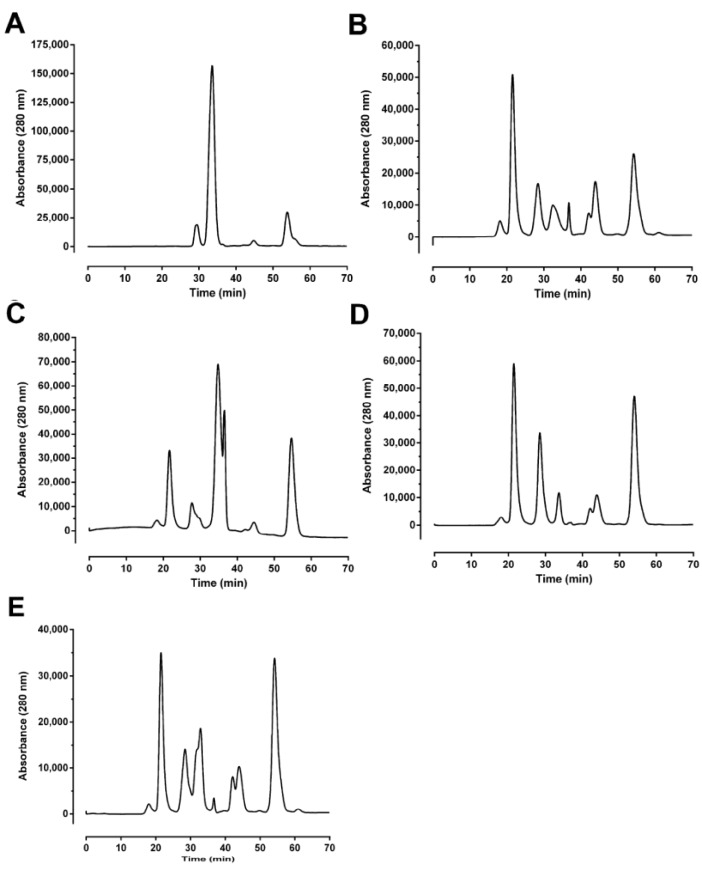
Size exclusion HPLC chromatograph of (**A**) *O. temporalis*; (**B**) *O. scutellatus*; (**C**) *O. microlepidotus*; (**D**) *O. scutellatus* (Saibai Island); and (**E**) *O. scutellatus* (Merauke) venoms run on a Superdex G-75 column equilibrated with ammonium acetate buffer (0.1 M, pH 6.8) at a flow rate of 0.5 mL/min.

Size-exclusion HPLC profiles of all venoms were obtained using a Superdex G-75 column (Figure 1). The profile of *O. temporalis* venom was simplistic when compared to the other taipan venoms with only one major peak and three minor peaks being apparent (Figure 1A). The major peak, which eluted at approximately 32 min, constitutes a substantial proportion of the venom. From the *O. temporalis* venom profile, there appears to be no peak eluting over the period of 20–24 min, which is a time period where a peak is observed in the profiles of the other venoms (Figure 1B–E) and has been previously shown to include the presynaptic neurotoxin in these venoms [7,8].

### 2.2. Reverse-Phase Chromatography

**Figure 2:**
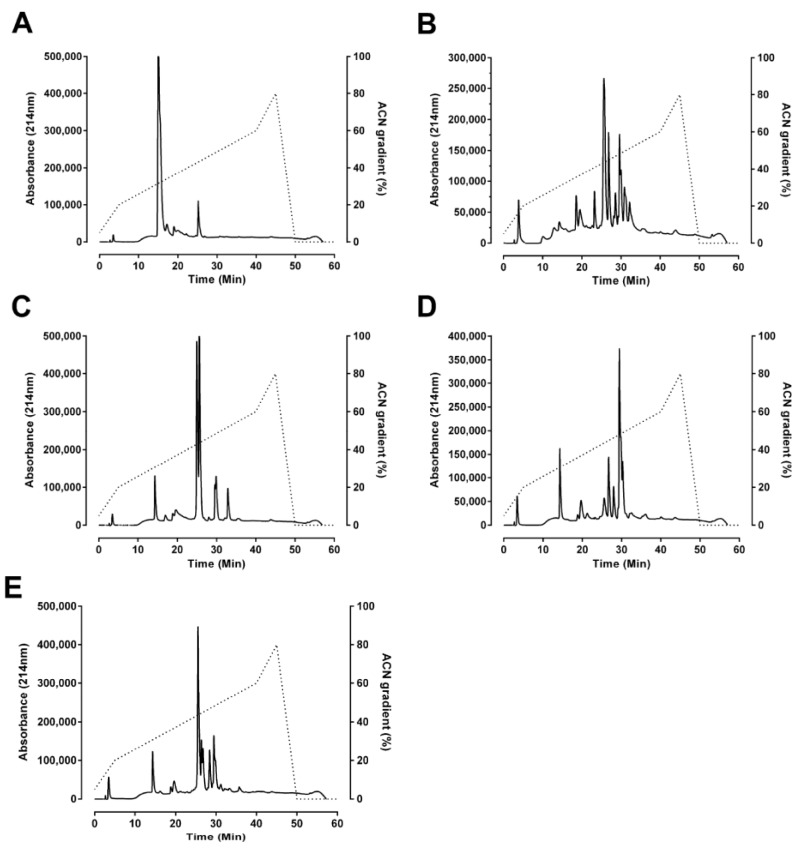
Reverse-phase HPLC chromatograph of (**A**) *O. temporalis*; (**B**) *O. scutellatus*; (**C**) *O. microlepidotus*; (**D**) *O. scutellatus* (Saibai Island); and (**E**) *O. scutellatus* (Merauke) venoms run on a Jupiter analytical C18 column equilibrated with 0.1% trifluoroacetic acid and gradient conditions of solvent B buffer (90% acetonitrile in 0.09% trifluoroacetic acid) at a flow rate of 0.2 mL/min.

Reverse-phase HPLC profiles of the taipan venoms were obtained using the Jupiter analytical C18 column (Figure 2). As per size-exclusion profiling, reverse-phase HPLC analysis indicated that *O. temporalis* venom is not a complex venom with a smaller number of peaks compared to the other taipan venoms (Figure 2A). The peak eluting between 15 min and 17 min represents a large proportion of the venom and is likely to contain short-chain postsynaptic neurotoxins.

### 2.3. SDS-PAGE Gel Experiments: Protein Molecular Size

**Figure 3:**
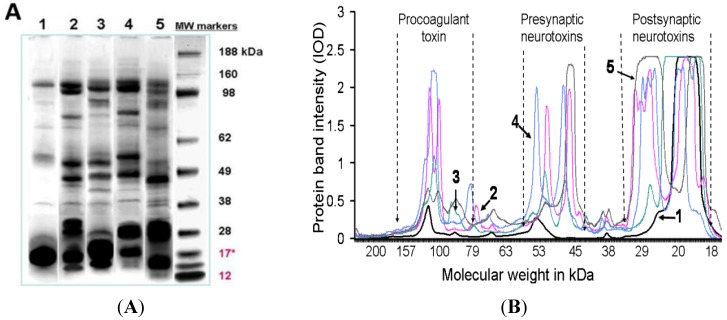
(**A**) SDS-PAGE analysis under non-reducing conditions (using a 4%–12.5% acrylamide gel plate); and (**B**) scan and computer estimations of the protein band molecular weights of the SDS-PAGE gel plate of taipan venoms (1) *O. temporalis*; (2) *O. scutellatus*; (3) *O. scutellatus* (Saibai Island); (4) *O. scutellatus* (Merauke); and (5) *O. microlepidotus*. MW indicates molecular standard markers. Note: The MW markers used in this electrophoresis are not reliable below 17 kDa molecular mass. The major taipan venom toxins have been highlighted in their approximate molecular weight position.

All venoms were analysed using SDS-PAGE under non-reducing conditions (Figure 3A). Scans of the gel and computer estimations of the protein band molecular weights of the gel plate showed again that *O. temporalis* venom is less complex than other taipan venoms with only light bands appearing in the 100–120 kDa and 50–53 kDa with the vast majority of the proteins found below 27 kDa (Figure 3B).

**Figure 4:**
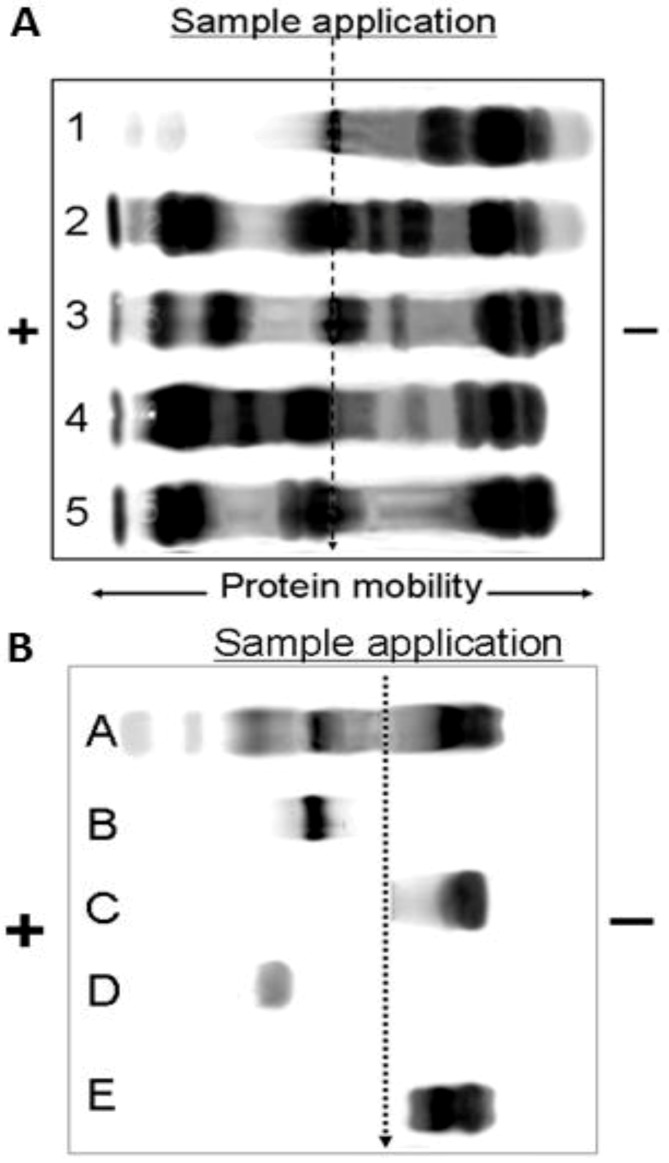
Protein mobility of (**A**) taipan venoms (1) *O. temporalis*; (2) *O. scutellatus*; (3) *O. scutellatus* (Saibai Island); (4) *O. scutellatus* (Merauke); and (5) *O. microlepidotus*; and (**B**) venom components (A) *O. scutellatus* venom; (B) Oscutarin; (C) Taicatoxin; (D) Taipoxin; (E) Postsynaptic taipan venom neurotoxins separated on agarose gel electrophoresis.

### 2.4. Agarose Gel Electrophoresis: Protein Mobility

From the protein mobility assay, it is clear that *O. temporalis* venom is comprised of proteins that mainly migrate towards the negative electrode at pH 8.6 indicating that they have positively charged proteins (Figure 4A). The *O. temporalis* venom appears to contain greatly reduced concentrations of several major toxins that are present in the other taipan venoms examined. This is evident by the light protein bands in positions where procoagulant toxins and presynaptic neurotoxins migrate (Figure 4B).

### 2.5. Lethality (LD_50_ Assay) in Mice

For *O. temporalis* venom, all mice in dose level groups 1.0× anticipated LD_50_ and lower survived. All mice in dose level groups 1.5× anticipated LD_50_ and higher did not survive. Therefore, the measured LD_50_ was calculated to be 0.075 mg/kg (*i.e*., 1.25× anticipated LD_50_ of 0.06 mg/kg). This was compared to *O. microlepidotus* venom, where the assay “end point” was not as clear. One out of four mice in dose level group 1.0× anticipated LD_50_ survived, two out of four mice in dose level 0.75× anticipated LD_50_ survived, and all four mice survived in dose level 0.5× anticipated LD_50_. All mice in the other higher venom dilution dose groups reached envenomation category 4 indicating they all had succumbed to the lethal effects of the *O. microlepidotus* venom. Therefore, the assay end point for *O. microlepidotus* venom was calculated to be 0.0225 mg/kg (*i.e*., 0.75× anticipated LD_50_ of 0.03 mg/kg).

### 2.6. Antivenom Efficacy in Mice

The results of the modified Mouse Protection Test (MPT) indicated CSL Taipan antivenom has similar ability to neutralise (*in vitro*) the lethal effects of both *O. temporalis* and *O. microlepidotus* venoms. The potency (one unit of activity) of the CSL Taipan antivenom in neutralising the effects of *O. microlepidotus* and *O. temporalis* venoms was calculated using simple direct proportion mathematics and using a definition of antivenom activity of one unit of activity being the amount of antivenom required to neutralise 10 µg (*i.e*., 0.01 mg) of venom. Using an end point dilution ratio for *O. microlepidotus* venom of 1/12.5 and for *O. temporalis* venom of 1/5, these calculations indicated that approximately the same quantity of CSL Taipan antivenom is required to neutralise *O. microlepidotus* venom and *O. temporalis* venom (*i.e*., 43 Units/mL and 32 Units/mL, respectively). It should be noted that the MPT bioassay is regarded as semi quantitative.

### 2.7. Chick Biventer Cervicis Nerve-Muscle Studies

**Figure 5:**
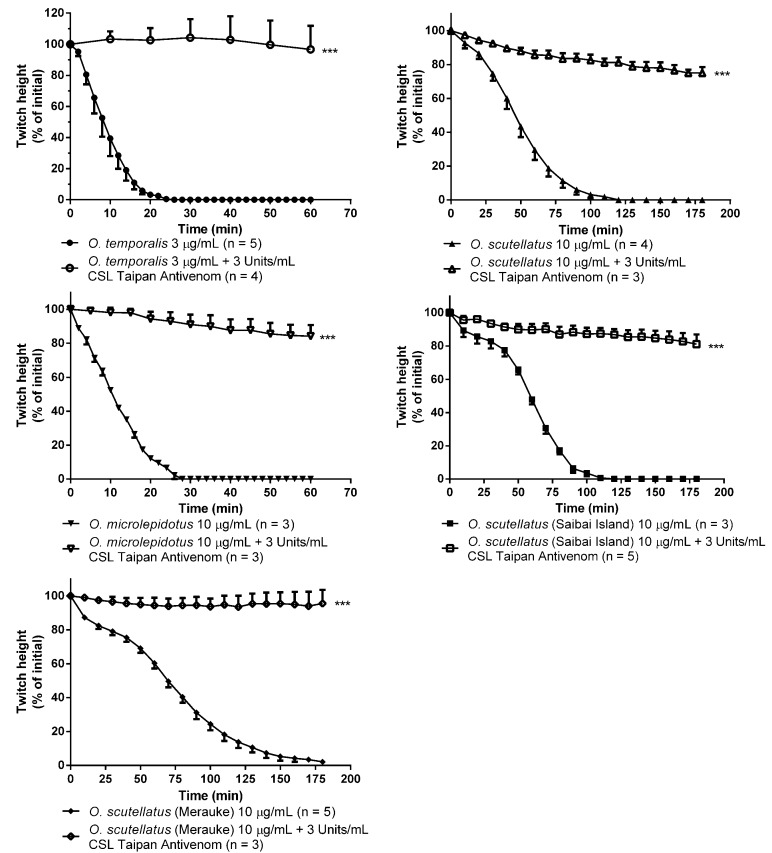
Effect of taipan venoms (3 µg/mL or 10 µg/mL) alone and in the presence of CSL Taipan antivenom (3 Units/mL) on nerve-mediated twitches of the chick biventer cervicis nerve-muscle preparation. *** *p* < 0.001, significantly different at either 60 or 180 min time point compared to venom alone (3 µg/mL or 10 µg/mL), Unpaired t test.

*O. temporalis* venom (1 µg/mL and 3 µg/mL) and *O. microlepidotus* venom (10 µg/mL) rapidly abolished indirect twitches of the chick biventer cervicis nerve-muscle preparation (Figure 5 and Table 1). Based on *t*_90_ values (*i.e*., time taken for 90% inhibition of the indirect twitches), at 10 µg/mL, *O. scutellatus*, *O. scutellatus* (Papuan Taipan-Saibai Island) and *O. scutellatus* (Papuan Taipan-Merauke) venoms were significantly less potent than *O. microlepidotus* venom (Table 1). However, at a concentration that was ten-fold less than the other taipan venoms tested (*i.e*., 1 µg/mL), *O. temporalis* venom showed strong neurotoxic effects with a *t*_90_ of 24.3 ± 4.1 min (*n* = 5) indicating that it is substantially more potent than all other taipan venoms tested (Table 1).

**Table 1:** *t*_90_ values (min) of venoms.

Species	Concentration (µg/mL)	*t*_90_ (min)
*O. temporalis*	1	24.3 ± 4.1 (5)
*O. temporalis*	3	15.1 ± 2.0 (5)
*O. microlepidotus*	10	21.2 ± 0.7 (3)
*O. scutellatus*	10	81.4 ± 6.6 (4) *
*O. scutellatus* (Saibai Island)	10	86.0 ± 2.5 (3) *
*O. scutellatus* (Merauke)	10	131.6 ± 10.0 (5) *

Data shown above as mean ± SEM. Number shown in parentheses indicates the number of preparations used from different animals. * *p* < 0.05 significantly different compared to *O. microlepidotus* 10 µg/mL, one way ANOVA followed by Bonferroni post-test.

**Figure 6:**
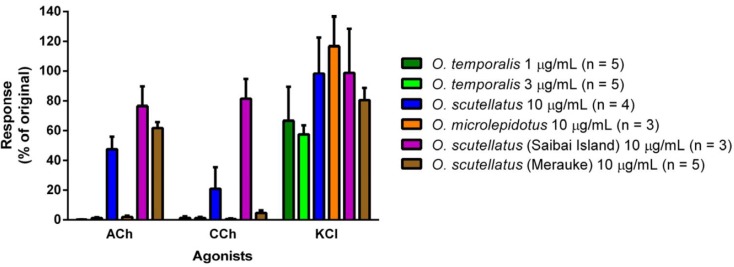
Effect of taipan venoms on chick biventer cervicis nerve muscle preparation responses to exogenous agonists; acetylcholine (ACh), carbachol (CCh) and potassium chloride (KCl).

Responses to ACh (1 mM) and CCh (20 µM) were abolished following exposure to *O. temporalis* venom (1 µg/mL and 3 µg/mL) and *O. microlepidotus* venom (10 µg/mL) indicating that these venoms contain postsynaptic neurotoxins, while the response to KCl (40 mM) was reduced (Figure 6). Agonist responses in the presence of *O. scutellatus*, *O. scutellatus* (Papuan Taipan-Saibai Island) or *O. scutellatus* (Papuan Taipan-Merauke) venoms were reduced, but not abolished, suggesting that these venoms contain less potent, or lower quantities, of postsynaptic neurotoxins (Figure 6).

Prior administration (10 min) of CSL Taipan antivenom (3 Units/mL) resulted in the neutralisation or a marked delay in the neurotoxic effects of all taipan venoms (3 µg/mL or 10 µg/mL; Figure 5).

## 3. Discussion

This study is the first examination of the neurotoxicity, lethality and biochemical properties of the venom of *O. temporalis*. Size-exclusion and reverse-phase HPLC profiles of *O. temporalis* venom revealed that the composition of the venom appears to be less complex compared to the other taipan venoms, with only one major peak and a few minor peaks. From previous studies in our laboratory, using the same reverse-phase HPLC conditions, short-chain postsynaptic neurotoxins typically elute at 15–17 min [10,24,25]. Therefore, based on this knowledge, it is highly likely that *O. temporalis* venom contains a substantial proportion of short-chain postsynaptic neurotoxins, as there is a large peak at this elution time in the reverse phase HPLC chromatogram. As mentioned previously, all taipan venoms have been found to contain short-chain postsynaptic neurotoxins, although the percentages of these neurotoxins range from 1.1% for taipan toxin 2 [11] to 9% of the whole venom for α-oxytoxin 1 [10]. Of considerable interest, is the fact that the size-exclusion profile of *O. temporalis* venom showed no peak between 20 min and 24 min, which is the usual elution time for the taipan presynaptic neurotoxins cannitoxin and taipoxin which have been isolated in our laboratory under similar size-exclusion HPLC conditions [7,8]. This suggests that *O. temporalis* venom does not contain a comparable presynaptic neurotoxin (*i.e*., approximately 45 kDa).

*O. temporalis* venom was analysed using SDS-PAGE and agarose gel electrophoresis. These data confirmed the lack of complexity of *O. temporalis* venom compared with the other taipan venoms. SDS-PAGE gels indicated that the majority of *O. temporalis* venom is comprised of lower molecular weight components. This was supported by the results of the agarose gel electrophoresis experiments with the venom appearing to contain only low (or no) procoagulant toxin or presynaptic neurotoxins. This substantial difference in the venom profile compared to the other taipan venoms could be attributed to a number of factors including that the venom was obtained, and pooled, from only two specimens. However, as these are the only two specimens in captivity, this will continue to be a limitation until more *O. temporalis* can be caught and studied.

The role of evolution and prey capture in the development of this simple venom profile is unclear. Examination of the scats from the two specimens of *O. temporalis* currently housed at Adelaide Zoo, as well as the gut contents from three preserved specimens held at WA Museum, indicated that *O. temporalis* feeds on small rodents and marsupials including; *Pseudomys* sp., *Sminthopsis* sp. and *Notomys alexis* (Personal communication B. Triggs). *O. microlepidotus* also feeds on rodents and marsupials such as; *Antechinomys laniger*, *Dasyurid* sp., *Mus musculus*, *Rattus sp.* and *R. villosissimus* [26], while *O. scutellatus* has been recorded to prey on *Isoodon macrourus*, *Perameles nasuta*, *Melomys* sp. *M. burtoni*, *M. cervinipes*, *Mus musculus*, *Rattus sordidus*, *R. tunneyi*, *Dasyurus hallicatus* [27] and has also been suggested to feed on birds [26].

*O. temporalis* venom rapidly abolished indirect twitches in the chick biventer cervicis nerve-muscle preparation at a concentration that was up to ten-fold less than the concentration required for the other taipan venoms. This suggests that *O. temporalis* immobilises its prey rapidly, which may prevent prey escaping and/or reduces the risk of damage to the snake from envenomed prey. This rapid neurotoxic effect suggests that the venom contains a high proportion of postsynaptic neurotoxins, which was supported by the fact that the venom also inhibited responses to exogenous nicotinic agonists, and also supports our observations regarding the reverse-phase HPLC profile of the venom. The rank order of potency in the skeletal muscle preparation was found to be: *O. temporalis* >> *O. microlepidotus* > *O. scutellatus* ≥ *O. scutellatus* (Saibai Island) > *O. scutellatus* (Merauke). Previous studies have investigated the neurotoxic activity of the other taipan venoms. Crachi *et al*. [20] determined that *O. microlepidotus* venom was significantly more potent than *O. scutellatus* and *O. s. canni* (Papuan Taipan, now O. s*cutellatus*) venoms at 10 µg/mL (*t*_90_ values 27 ± 3 min, 42 ± 3 min and 48 ± 5 min, respectively), thus supporting the results of this present study. Crachi *et al*. [20] also showed that all three venoms inhibited responses to ACh and CCh, without effecting KCl responses, indicating that taipan venoms contain postsynaptic neurotoxins, a fact again mirrored in this current study. The present study also investigated the efficacy of CSL Taipan antivenom. Prior addition of CSL Taipan antivenom prevented the neurotoxic effects of *O. temporalis* venom. Similarly, CSL Taipan antivenom either delayed or prevented the inhibitory effects of the other taipan venoms. These results confirm the work of Crachi *et al*. [20] and also indicate that CSL taipan antivenom is likely to be clinically effective against the neurotoxic components of *O. temporalis* venom. However, whether this “protective” effect can be translated to a human envenoming is uncertain.

The present study also examined the lethality of *O. temporalis* and *O. microlepidotus* venoms in a murine model. Based on murine LD_50_ values, *O. microlepidotus* venom was found to be more “lethal” compared to *O. temporalis* venom (0.0225 mg/kg *vs*. 0.075 mg/kg, intraperitoneally (i.p.)). Previous studies on taipans have found that they are some of the most venomous snakes in Australia, with Broad *et al*. [28] showing the *O. microlepidotus* (previous known as *Parademansia microlepidotus*) to be the most deadly, with an LD_50_ (i.p., in saline) value of 0.025 mg/kg. *O. scutellatus* was ranked third with an LD_50_ value of 0.099 mg/kg. Therefore, it would seem that *O. temporalis* venom is more lethal than *O. scutellatus* venom, but less lethal than the *O. microlepidotus* in the murine model.

Although lethality (LD_50_) studies give researchers important information regarding venoms and toxins, they do have their limitations, in the sense that they measure “quantity” *i.e*., amount of venom/toxin that kills 50% of mice over a certain period of time and do not take into consideration the time frame of the venom/toxin activity [29]. Usually whole venom is used which limits identification of the type(s) of toxins contributing to the final value and the level of toxicity of each family of toxin [30]. Thus, potentially it is plausible to have a very “lethal” venom (*i.e*., low LD_50_ values) that takes a long time to show its lethal effects *in vivo* but may show more rapid effects using an *in vitro* preparation due to the presence of postsynaptic neurotoxins [29].

A preliminary study of the procoagulant activity of the taipan venoms was also conducted using a visual assay (data not shown). At concentrations of 10–1000 ng/mL, *O. temporalis* venom appeared to have limited procoagulant activity compared to the activity of the other taipan venoms. Even then, the concentrations required to induce this activity were markedly higher than the upper range of Australian elapid venom levels reported in envenomed humans (e.g., *Pseudonaja* sp. 50–100 ng/mL; [31]). As mentioned previously taipan venom (*O. scutellatus*) has been shown to contain a prothrombin activator called oscutarin [16,17]. Interestingly, more recent work has shown that the procoagulant activity of *O. scutellatus* venom is markedly less potent than some other Australian elapids such as *Pseudonaja* sp. [32]. However, more research is needed to fully characterise any procoagulant toxins present in *O. temporalis* venom.

## 4. Experimental Section

### 4.1. Venom

*O. temporalis* venom was obtained from two live specimens (a male and female) held in captivity at Adelaide Zoo in Adelaide, South Australia. The snakes were maintained separately, at 25–29 °C, on paper substrate in wooden glass fronted cages. The male was fed adult mice on 7 to 14 day intervals and the female was fed rat pups at 7 to 14 day intervals. For the first three milkings, the snakes were milked using method two [33] and for the fourth and fifth milking, method three was used [33].

*O. scutellatus*, *O. microlepidotus* and *O. scutellatus* (Papuan Taipan-Saibai Island) venoms were obtained from venom production snakes kept at Venom Supplies Pty Ltd, Tanunda, South Australia. In the case of the latter, the venom was obtained from a single snake specimen. These snakes were kept either on paper substrates in wooden cages with partial mesh tops or plastic tubs. They were maintained at a gradient which ranged between 17 °C and 35 °C. They were all fed on rats of various sizes on a 7–14 day basis and were milked using method two [33]. *O. scutellatus* (Papuan Taipan-Merauke) venom was obtained from Duncan Macrae (Bali, Indonesia). The milking method used was method two [33].

After the venom was collected, it was immediately snap frozen with dry ice, transferred into a −20 °C freezer and then lyophilised at a later date. After lyophilisation, the venom was stored at 4–8 °C until required. To prepare the venom samples for this study and to ensure consistency of venom samples used across the experiments, the venom was pooled (where required) and reconstituted using 0.15 M NaCl solution to a concentration of 20 mg/g of solution. From this solution, aliquots of 500 mg were pipetted into 2 mL vials, frozen at −80 °C and then lyophilised to produce individual samples of 10 mg of each taipan venom, to be dispersed between researchers.

### 4.2. Chromatography

Both size-exclusion and reverse-phase chromatography separations were performed using a Shimadzu (Kyoto, Japan) high-performance liquid chromatography (HPLC) system (LC-10ATvp) pump and SPD-10AVP detector.

#### 4.2.1. Size-Exclusion HPLC

Freeze-dried venom was dissolved in Milli-Q water (Millipore Corporation, Billerica, MA, USA), to give a stock solution of 500 µg/mL. Samples were vortexed briefly to aid dissolving and were then subjected to centrifugation at 12,000 rpm for 8 min. The supernatant was then filtered using 0.2 µm Supor^®^ Membrane Acrodisc^®^ Syringe filters (PALL Life Science, Ann Arbor, MI, USA) and the resulting solution was transferred to new microtubes (Axygen Inc, Union City, CA, USA) and re-centrifuged as previously described. Supernatant from this second centrifugation was then applied to a Superdex G-75 column (13 µm; 10 mm × 300 mm; GE Healthcare, Little Chalfont, Buckinghamshire, UK) equilibrated with ammonium acetate buffer (0.1 M Ammonium acetate, pH 6.8). The sample was eluted at a flow rate of 0.5 mL/min and was monitored at 280 nm.

#### 4.2.2. Reverse-Phase HPLC

Freeze-dried venom was dissolved in Mill-Q water (Millipore Corporation, Billerica, MA, USA) to give a stock solution of 1 mg/mL. Samples were vortexed briefly to aid dissolving and were then centrifuged at 6000 rpm for 5 min. Samples were then applied to Phenomenex (Torrance, CA, USA) Jupiter analytical C18 column (150 mm × 2 mm; 5 µm; 300 Å) after equilibrating with 95% solvent A (0.1% trifluoroacetic acid, TFA) and 5% solvent B (90% acetonitrile (ACN) in 0.09% TFA). The samples were then eluted with the following gradient conditions of solvent B at a flow rate of 0.2 mL/min; 0% to 20% over 5 min, 20% to 60% between 5 min and 40 min, and then 60% to 80% between 40 min and 45 min, and finally 80% to 0% between 45 min and 50 min. The eluant was monitored at 214 nm.

### 4.3. SDS-PAGE Gel Experiments: Protein Molecular Size

For SDS-PAGE gel experiments venoms were diluted to 1 mg/mL with saline (0.15 M NaCl). Samples (10 µL) were then further diluted by adding 4 µL of SDS-PAGE sample buffer and then incubated at 70 °C for 10 min. All samples were non-reduced, preserving the tertiary structure of the proteins. Following incubation, 10 µL of the venom-SDS buffer solution was loaded onto a SDS-acrylamide gel plate and run in a 1:20 dilution of MOPS running buffer which was made from a concentrated stock buffer solution. Gels were then stained with 0.1% (*w*/*v*) Coomassie Brilliant Blue R-250 and subsequently de-stained in a mixture of acetic acid-water-methanol (1:5:5). The stained SDS-PAGE and agarose gel protein bands were then evaluated using Gel-Pro Analyzer (Media Cybernetics, Silver Spring, MD, USA) computer imaging software (Version 3.0 for Windows 1997).

### 4.4. Agarose Gel Electrophoresis: Protein Mobility

The electrophoretic mobility of proteins found in the venoms was investigated using a method previously described in Madaras *et al*. [34]. All venoms (1 µL of a 30 mg/mL stock venom solution) were individually loaded into agarose gel plate sample wells. Electrophoresis was performed using 50 mM sodium barbitone buffer (pH 8.6 at ~2 Vcm^2^). Following electrophoresis the gel was stained as via the same method described above for SDS acrylamide gels and was also evaluated via the same process.

### 4.5. Mice: LD_50_ Determination

LD_50_ determination was carried out using the conventional mice lethality assay modified to meet Animal Ethics Committee (AEC) requirements. Prior to the assay being performed an anticipated LD_50_ value for each venom was derived from published literature and knowledge of LD_50_ values for similar venoms [28]. Fresh stock venom solutions were prepared in 0.9% sterile saline to provide solutions of 4, 3, 2, 1.5, 1.0, 0.75 and 0.5 times the anticipated LD_50_ value (*i.e*., 7 different doses), to cover a range of doses either side of the anticipated LD_50_ value. To minimise non-specific binding of the venom components, only polypropylene and polycarbonate test tubes and containers were used.

Twenty-eight, male Balb/c mice (18–28 g) were randomly allocated to seven venom dose groups, with four mice per venom dose (as per above). The mice in each dose group were injected with 0.2 mL (i.p.) of the appropriate venom solution and then observed closely over the following eight hours for signs of envenoming. At regular intervals, animals were subjectively scored on a graded scale of 1 to 4 as to the severity of signs of envenoming (*i.e*., ruffled coat, huddling, dyspnoea, reduce responsiveness and paralysis). Mice that were unaffected were given a score of 1, while mice that were severely affected (almost moribund, near death) were given a score of 4. At this point the animal was humanely euthanized by cervical dislocation. Mice that were still alive at the end of the 8 h time period were also humanely euthanized in this way.

At the end of the 8 h observation period the venom dose group of mice receiving the highest venom dose in which at least one animal “survived” was regarded as the end point of the bioassay. e.g., if in the dose level group of 1.5 times anticipated LD_50_ two out of the 4 mice survived then the LD_50_ value of the test venom would be deemed to be 1.5× anticipated LD_50_. Statistical analysis of this data cannot be meaningfully undertaken as the number of animals used is limited to four per dose level group. This is a requirement of the AEC which approved the bioassay. Thus the assay result must be regarded as a semi quantitative value.

### 4.6. Mice: Antivenom Efficacy

Antivenom efficacy was determined by the conventional mouse protection test (MPT) modified to meet AEC requirements. Two groups, each of twenty-eight Balb/c mice (22.9 ± 1.5 g) were randomly allocated to 7 groups of 4 mice. Stock solutions of *O. temporalis* and *O. microlepidotus* venoms were prepared in sterile 0.9% saline at a concentration of 5× LD_50_, as determined above. Serial dilutions of CSL Taipan antivenom (349 Units/mL), were also prepared in sterile 0.9% saline to provide 7 dilutions of antivenom: 1/5, 1/10, 1/15, 1/22.5, 1/33.8, 1/50.6 and 1/76. The 5× LD_50_ venom solution (1 mL) was mixed with each of the antivenom solutions (1 mL) and incubated for 30 min at 37 °C in a water bath to allow for venom neutralisation, then 0.2 mL of each solution was injected i.p. into four mice. An additional 2 positive (*i.e*., venom only) and 2 negative (*i.e*., antivenom only) control animals were included making a total of 32 mice for each venom. Mice were then observed closely over the following 8 h for signs of envenoming and subjectively scored on a scale of 1 to 4 (as per above). At the end of the observation period the highest antivenom dilution group with mice surviving was regarded as the assay end point group. Mice still alive at the end of the 8 h time period were humanely euthanized as previously described.

### 4.7. Chick Biventer Cervicis Nerve-Muscle Preparation

Male chickens (4–10 days old) were killed by CO_2_ inhalation and exsanguination and the biventer muscles with associated nerves were dissected. Each tissue was then attached to a wire holder and placed in a 5 mL organ bath, under a resting tension of 1 g, filled with physiological salt solution of the following composition (mM): NaCl, 118.4; NaHCO_3_, 25; glucose, 11.1; KCl, 4.7; MgSO_4_, 1.2; KH_2_PO_4_, 1.2; CaCl_2_, 2.5. The organ baths were bubbled with carbogen (95% O_2_, 5% CO_2_) and maintained at a temperature of 34 °C. The preparation was stimulated using a Grass stimulator (0.1 Hz, 0.2 ms, supramaximal voltage; *i.e*., approx. 12–18 V) and the twitches recorded on a PowerLab system via a Grass FT03 transducer. The tissues were equilibrated for 10–15 min after which d-tubocurarine (10 µM) was added. The subsequent abolition of twitches indicated that only the somatic nerve was being stimulated. Repeated washing of tissues with physiological solution was performed until twitch height was restored. In the absence of nerve stimulation, responses to acetylcholine (ACh, 1 mM; 30 s), carbachol (CCh, 20 µM; 60 s) and potassium chloride (KCl, 40 mM; 30 s) were obtained. Electrical stimulation was then recommenced for 30 min, as above, prior to the addition of venom (1–10 µg/mL depending on the potency of the venom). Venoms were left in contact with the tissue until twitches were completely abolished or for at least 3 h. At this point, stimulation was ceased and responses to ACh, CCh and KCl were retested as previously described.

To examine the *in vitro* effectiveness of antivenom, the above protocol was followed except CSL Taipan antivenom (3 Units/mL) was added to the organ bath 10 min prior to the addition of venom (3 µg/mL or 10 µg/mL) and tissues were left in contact with venom/antivenom for either 1 h (*O. temporalis* and *O. microlepidotus*) or 3 h (*O. scutellatus*; Australian coastal taipan, Papuan Taipan-Saibai Island and Papuan Taipan-Merauke).

### 4.8. Chemicals and Drugs

For neurotoxicity and HPLC experiments the following chemicals and drugs were used: acetylcholine chloride (ACh, Sigma-Aldrich, St. Louis, MO, USA), carbamylcholine chloride (carbachol CCh, Sigma-Aldrich, St. Louis, MO, USA), d-tubocurarine (Sigma-Aldrich, St. Louis, MO, USA), ammonium acetate (Sigma-Aldrich, St. Louis, MO, USA), potassium chloride (KCl, Ajax Chemicals Pty Ltd, Sydney, Australia); trifluoroacetic acid (TFA, Auspep, Melbourne, Australia) and acetonitrile (ACN, Merck, Darmstadt, Germany). For SDS-PAGE and agarose gel electrophoresis experiments NuPAGE ready-made 4%–12% (1.5 mm thick) SDS-acrylamide gels, MOPS-SDS running buffer and SDS sample buffer were purchased from Invitrogen Pty Ltd while agarose was purchased from Amersham Biosciences Pty Ltd. All experiments involving antivenom used CSL Taipan antivenom (CSL Ltd., Melbourne, Australia) (Batch number: B0548-06401 Expiry: 10/12).

### 4.9. Data Analysis

For neurotoxicity experiments using the chick biventer cervicis nerve-muscle preparation twitch height, measured at regular time intervals, was expressed as a percentage of the twitch height prior to venom addition. Contractile responses to exogenous ACh, CCh and KCl were expressed as percentages of their initial responses. One-way analysis of variance (ANOVA) followed by Bonferroni post-test was used to compare the inhibitory effects of venoms (Prism 5.0, GraphPad Software, San Diego, CA, USA). For antivenom studies, carried out in the chick biventer cervicis nerve muscle preparation, unpaired t tests were used to analyse whether there was statistical significant differences (*p* < 0.05) between venom alone (either 3 µg/mL or 10 µg/mL) and CSL Taipan antivenom (3 Units/mL) plus venom (either 3 µg/mL or 10 µg/mL). Where “*n*” indicates the number of individual chick biventer cervicis nerve-muscle preparations used and error bars in figures represent the standard error of the mean (*i.e*., s.e.m.).

## 5. Conclusions

From this first investigation of *O. temporalis* venom, we now have greater insights into the composition and activity of the venom. The major finding is that the venom contains a substantial proportion of postsynaptic neurotoxins, a low percentage of procoagulant toxins (or toxins with low potency) and may also contain a non-typical presynaptic neurotoxin. However, additional studies isolating and characterising individual components of *O. temporalis* venom are required, such research is already underway. From this study we can conclude that if a person was bitten by *O. temporalis*, neurotoxicity is a clinical symptom that may be evident following envenoming. Therefore, this snake should be viewed as potentially dangerous to humans and domestic animals, and should be approached with care.
